# Immunosuppressant-Induced Alteration of Gut Microbiota Causes Loss of Skeletal Muscle Mass: Evidence from Animal Experiments Using Mice and Observational Study on Humans

**DOI:** 10.3390/jcm14051628

**Published:** 2025-02-27

**Authors:** Mitsuru Tomizawa, Shunta Hori, Tatsuo Yoneda, Fumisato Maesaka, Sayuri Onishi, Takuto Shimizu, Kenta Onishi, Yosuke Morizawa, Daisuke Gotoh, Yasushi Nakai, Makito Miyake, Kazumasa Torimoto, Nobumichi Tanaka, Kiyohide Fujimoto

**Affiliations:** 1 Department of Urology, Nara Medical University, 840 Shijo-cho, Kashihara 634-8522, Nara, Japan; tomimit.com@gmail.com (M.T.); horimaus@gmail.com (S.H.); uro-yone@naramed-u.ac.jp (T.Y.); mae_fumi0107@yahoo.co.jp (F.M.); sayuri3@naramed-u.ac.jp (S.O.); takutea19@gmail.com (T.S.); kenzmedico0912@yahoo.co.jp (K.O.); tigers.yosuke@gmail.com (Y.M.); dgotou@gmail.com (D.G.); nakaiyasusiuro@live.jp (Y.N.); makitomiyake@yahoo.co.jp (M.M.); torimoto@naramed-u.ac.jp (K.T.); sendo@naramed-u.ac.jp (N.T.); 2 Department of Prostate Brachytherapy, Nara Medical University, 840 Shijo-cho, Kashihara 634-8522, Nara, Japan

**Keywords:** kidney transplantation, gut microbiota, muscle mass, immunosuppressant, *Akkermansia*, tacrolimus, prednisolone

## Abstract

**Background/Objectives**: The number of older adults requiring a kidney transplant (KT) is increasing; hence, postoperative sarcopenia prevention is necessary. KT recipients require permanent oral immunosuppressants (ISs), and the gut microbiota (GM) plays a role in various systemic diseases. However, few studies have evaluated post-kidney transplantation frailty and the associations among ISs, GM, and muscle mass alterations. Therefore, we investigated the effects of ISs on GM and skeletal muscle mass in mice and human KT recipients. **Methods:** Mice were treated with six different ISs, and their skeletal muscle mass, GM diversity, and colonic mucosal function were assessed. Human KT recipients and donors were monitored before and after surgery for 1 year, and GM diversity was evaluated before and 1 month after surgery. **Results**: The abundance of *Akkermansia*, crypt depth, and mucin 2 expression were lower in tacrolimus- and prednisolone-treated mice. The psoas muscle volume changes at 1 month and 1 year after surgery were lower in KT recipients than in donors. Furthermore, the beta diversity was significantly different between the operative groups (*p* = 0.001), and the KT group showed the lowest Shannon index. **Conclusions**: The findings of this study indicate potential links among ISs, GM, and muscle mass decline. Further investigation is required to improve therapeutic strategies and patient outcomes.

## 1. Introduction

Kidney transplantation is the best treatment option for patients with end-stage renal disease (ESRD) [1,2,3,4]. Owing to recent improvements in available immunosuppressants (ISs), kidney transplantation has become the best treatment for ESRD in older adults [5,6]. In Japan, the number of recipients between the ages of 60 and 79 years was higher in 2015 than in 2007 [7] and continues to increase. Considering that age may influence the suitability of patients for kidney transplant (KT), age has been recommended as an additional consideration along with other comorbidities, such as frailty, by the Kidney Disease Improving Global Outcomes clinical practice guidelines [8]. Therefore, preoperative frailty assessment and postoperative muscle mass maintenance are important for KT recipients. The prevalence of frailty is high among all KT recipients, including preemptive and younger recipients [9], and preoperative frailty is associated with poor postoperative outcomes, including increased mortality [10], longer hospital stay durations following kidney transplantation [11], and increased complications [12]. However, few studies have evaluated post-kidney transplantation frailty over time, and conflicting and controversial postoperative frailty results have been reported. These results include the following: postoperative frailty initially worsened but improved within 3 months [13], worsened 2.5 years after kidney transplantation despite shorter-term improvements [14], or muscle mass showed consistent decline after kidney transplantation over a 1-year follow-up [15].

The involvement of the gut microbiota (GM) through the gut–muscle axis has been recently reported in various systemic diseases [16]. In KT recipients, permanent oral administration of multiple ISs is required to suppress rejection; however, ISs may alter the GM [17]. Nevertheless, few studies have evaluated the associations among ISs, GM, and muscle mass. Therefore, in this study, we evaluated the effects of various ISs on GM and muscle mass in mice, assessed the body composition of living KT recipients and donors over a 1-year period, and monitored the chronological change rate and GM composition.

## 2. Materials and Methods

### 2.1. Study Design

In this study, we conducted animal experiments using mice and an observational study on living human KT recipients and donors at our hospital. The animal experiments were approved by the Animal Facility Committee (Approval No. 13177), and the clinical study was approved by the Institutional Review Board for Clinical Studies (Medical Ethics Committee ID: 2749) at Nara Medical University. All methods are reported in accordance with the ARRIVE guidelines (https://arriveguidelines.org [accessed on 27 February 2025]). The clinical study was conducted in compliance with the study protocol and provisions of the Declaration of Helsinki (2013). Informed consent was obtained from all recipients and donors after the study was explained to them.

### 2.2. Animals and Experimental Design

**Figure 1:**
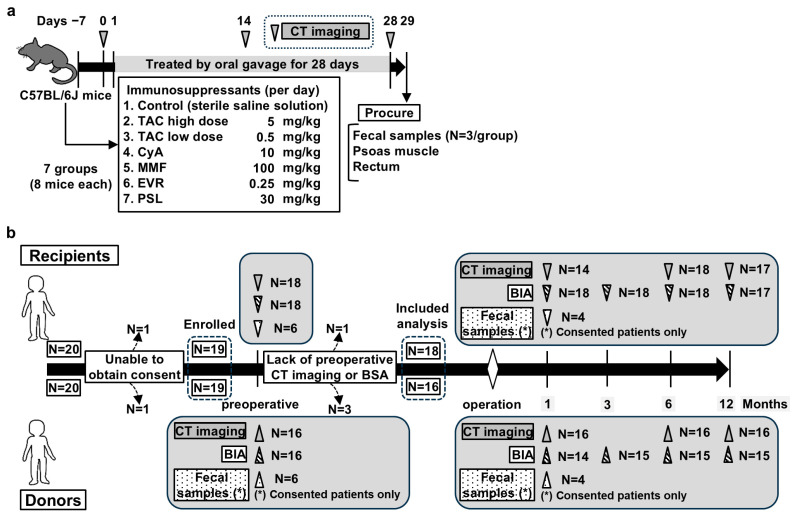
(**a**) A schematic illustration of the mouse experiment workflow. The mice were randomly divided into seven groups (n = 8/group), and treatments were administered through gastric gavage once daily for 28 consecutive days. The mice were subjected to computed tomography (CT) imaging on days 0, 14, and 28. The mice were euthanized on the day after the final CT imaging, and blood, psoas muscle, rectum, and fecal samples were harvested. Feces were collected from three mice/group. (**b**) The workflow of the observational study on human kidney transplant (KT) recipients and donors. In total, 20 consecutive living donor kidney transplantations were performed at our institute, and 19 recipients and 19 donors who consented to this study were enrolled. Patients who could not comply with preoperative CT or bioelectrical impedance analysis (BIA) were excluded (one recipient and three donors). Finally, the data of 18 recipients and 16 donors were included in the analyses. Post-surgery, the patients were subjected to CT imaging at 1, 6, and 12 months and BIA at 1, 3, 6, and 12 months. Missing data at any point were allowed, and only the obtained data were analyzed. Fecal samples were collected only from consenting patients. The number of data analyzed at each point is indicated to the right of the arrow.

In total, 56 C57BL/6J male mice (6 weeks old) were purchased from Oriental Bio Service (Kyoto, Japan) and housed at 23 ± 3 °C under 50% ± 20% humidity. The details of the reagents and their preparation are provided in the Supplementary Materials, and a schematic of the experimental design is illustrated in Figure 1a. After acclimating to laboratory conditions for 1 week, the mice were randomly divided into seven groups (n = 8 mice/group): control (sterile saline solution), tacrolimus (TAC) high-dose (5 mg/kg/day), TAC low-dose (0.5 mg/kg/day), cyclosporine A (CyA) (10 mg/kg/day), everolimus (0.25 mg/kg/day), mycophenolate mofetil (100 mg/kg/day), and prednisolone (PSL) (30 mg/kg/day). The mice were administered the respective solutions through gastric gavage once daily for 28 consecutive days. The mice were subjected to computed tomography (CT) imaging on days 0, 14, and 28 using CosmoScan FX (Rigaku Corporation, Tokyo, Japan) and euthanized the day after the final CT imaging. All mice were euthanized by exsanguination under anesthesia with isoflurane, and tissues (psoas muscle, rectum, and fecal samples) were harvested. Fecal samples were collected from three mice per group. All procedures were performed on mice under anesthesia with isoflurane, and all efforts were made to minimize suffering, including providing sufficient sedation and reducing the treatment time.

### 2.3. Measurement of Psoas Muscle Using CT Images and Pathological Specimens

The cross-sectional area of the psoas muscle was assessed from each CT slice at the L3, L4, and L5 pedicle levels using a SYNAPSE SAI viewer (Fujifilm Corporation, Tokyo, Japan). The rate of change in each cross-sectional area was evaluated for each mouse from day 0. The harvested psoas muscles were fixed in 10% formalin, embedded in paraffin, sliced into 5 μm thick sections, and stained with hematoxylin and eosin. The myocyte cross-sectional length was assessed for 15 fibers/mouse in high-power fields at 400× magnification using ImageJ software 1.54 f [18].

### 2.4. Fecal Sample Collection, DNA Extraction, 16S rRNA Gene Sequencing, and Processing

The procedures for fecal sample collection, DNA extraction, 16S rRNA gene sequencing, and data processing are provided in the Supplementary Materials, and an overview of the data processing procedure is shown in Figure S1.

### 2.5. Evaluation of Colonic Mucosa

The procured rectal tissues were fixed in 10% formalin, embedded in paraffin, sliced into 5 μm thick sections, and stained with Alcian blue. The mucosal crypt depth was measured by analyzing at least 25 well-oriented crypts from eight mice/group. The crypt depth was defined as previously described [19] and measured using ImageJ software 1.54 f [18]. Immunohistochemical (IHC) staining was performed for mucin-2 (MUC2), as previously described [20]. The IHC and data collection procedures are provided in the Supplementary Materials.

### 2.6. KT Recipients and Donors: Patient Selection, Data Collection, Study Design, and Perioperative Protocol

A flow diagram of patient selection and study design is presented in Figure 1b. The primary endpoint was the change rate in the postoperative psoas major muscle measured using CT imaging 1 year after surgery from the preoperative value. The secondary endpoints were changes in the postoperative body composition measured using CT images and bioelectrical impedance analysis (BIA) at each point (1, 3, 6, and 12 months post-surgery) and changes in the postoperative GM from the preoperative data. In total, 20 consecutive living donor kidney transplantations were performed between December 2020 and August 2022 at our institution, and 19 recipients and 19 donors who consented to participate in this study were enrolled. Patients who could not undergo preoperative CT or BIA were excluded (one recipient and three donors); therefore, 18 recipients and 16 donors were included in the analysis. These patients underwent CT imaging at 1, 6, and 12 months and BIA at 1, 3, 6, and 12 months postoperatively. With the exception of the first month after surgery, data from patients lacking clinical stability—defined as those requiring hospitalization within a month except for transplant kidney biopsy—were excluded. However, the data of only one recipient who lacked clinical stability 1 year after surgery were excluded. Missing data at each point were permitted, and only the obtained data were analyzed. The number of data analyzed at each time point is shown in Figure 1b. Fecal samples were only collected from patients who provided consent. GM analysis was performed using the same procedure as that used for the mouse fecal analysis. The procedures for measuring body composition are described in the Supplementary Materials. Briefly, the obtained unenhanced CT images were analyzed using the Volume Analyzer SYNAPSE VINCENT image analysis system (Fujifilm Medical, Tokyo, Japan), and body composition was measured using multifrequency BIA with the InBody S20^®^ analyzer (Biospace, Tokyo, Japan), as previously described [21]. To evaluate the changes in body composition, the preoperative change rate was calculated individually for each patient from the obtained data using the following formula:

Postoperative value/Preoperative value × 100 − 100 (%).

The post-transplant maintenance immunosuppressants used were primarily oral calcineurin inhibitors (TAC or CyA), mycophenolate mofetil, and PSL, with everolimus added at the discretion of the attending physician. Basiliximab was intravenously administered on the day of the KT and 4 days later as an induction immunosuppressant. In ABO-incompatible KT, rituximab was intravenously administered on the 14th day and 1 day before KT.

Both the recipients and donors were administered a single dose of cefazolin sodium as a perioperative antibiotic. All recipients received oral sulfamethoxazole and trimethoprim for at least 1 year to prevent pneumocystis pneumonia.

### 2.7. Statistical Analysis

Statistical analyses and figure plotting were performed using GraphPad Prism 10.1.1 (GraphPad Software, San Diego, CA, USA). Comparisons between the two groups were performed using the Mann–Whitney *U* test. A *p*-value < 0.05 was considered statistically significant. For the animal experiments, the control and IS treatment groups were compared using the Mann–Whitney *U* test without correction for multiple comparisons. Statistical differences in the beta diversity of the GM were analyzed using permutational multivariate analysis of variance with Quantitative Insights into Microbial Ecology 2. Benjamini−Hochberg adjustment was applied for comparisons between more than two groups. A *p*-value < 0.05 was considered statistically significant and the *q*-value was noted. Statistical differences in the alpha diversity of the GM were analyzed using the Mann–Whitney *U* test or the Kruskal–Wallis test and Dunn’s method with a false discovery rate (FDR) approach (two-stage step-up method of Benjamini, Krieger, and Yekutieli). Statistical significance was set at an FDR value (*q*-value) of < 0.1. Statistical differences in the data obtained from the Phylogenetic Investigation of Communities by Reconstruction of Unobserved States 2 (PICRUSt2) analysis were divided into two groups using the Mann–Whitney *U* test with the FDR approach. Owing to the large amount of data analyzed, differences were considered statistically significant at a *q*-value < 0.01.

## 3. Results

### 3.1. Psoas Muscle Cross-Sectional Area and Myocyte Cross-Sectional Length in Mice

**Figure 2:**
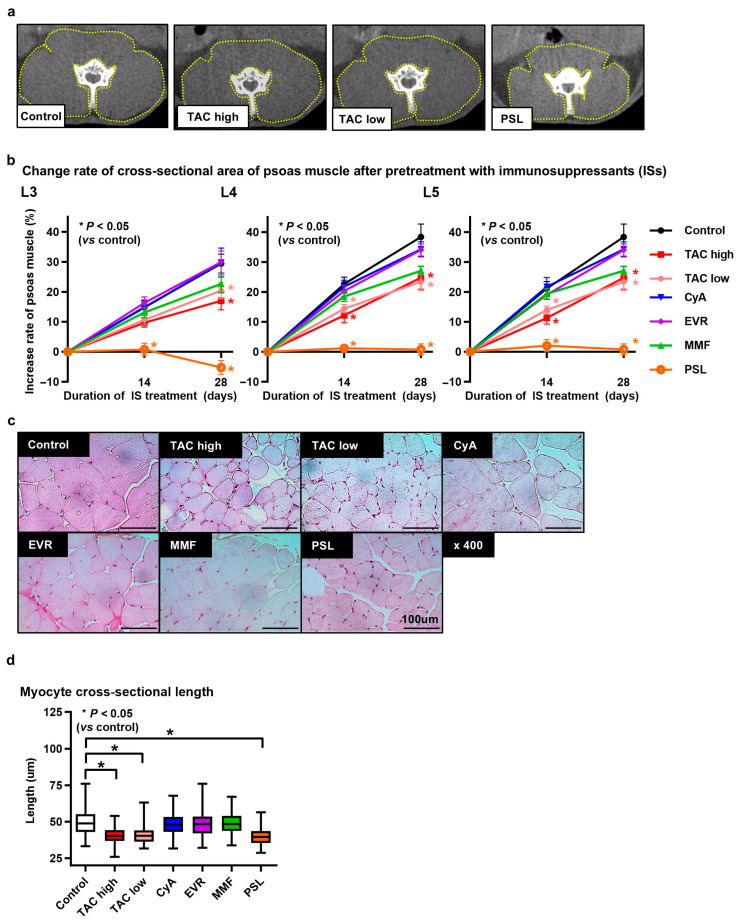
(**a**) Representative computed tomography (CT) images at the L5 pedicle level after being treated with various immunosuppressants. (**b**) Change rate of psoas muscle cross-sectional area in each group at the L3, L4, and L5 pedicle levels. (**c**) Representative images of hematoxylin and eosin (HE)-stained myocytes from each group following treatment with various immunosuppressants. Scale bar = 100 μm. (**d**) Comparison of myocyte cross-sectional length of each group after treatment with various immunosuppressants.

At all levels, the rate of psoas muscle cross-sectional area increase in the control group was higher than those in the TAC high-dose, TAC low-dose, and PSL groups on day 28 (L3: *p =* 0.049, *p =* 0.049, *p <* 0.001; L4: *p =* 0.04, *p =* 0.04, *p <* 0.001; L5; *p =* 0.001, *p =* 0.01, *p <* 0.001, respectively) (Figure 2a,b). The PSL group showed the lowest rate of increase among all groups. The myocyte cross-sectional length in the control group was longer than those in the TAC high-dose, TAC low-dose, and PSL groups (*p <* 0.001, *p <* 0.001, *p <* 0.001, respectively) (Figure 2c,d). Based on these results, the TAC high-dose, TAC low-dose, and PSL groups were grouped into the poor muscle development group (PMD group) and the other groups into the muscle development group (MD group).

### 3.2. Differences in GM Composition and Metabolic Function Between the MD and PMD Groups After IS Treatment

**Figure 3:**
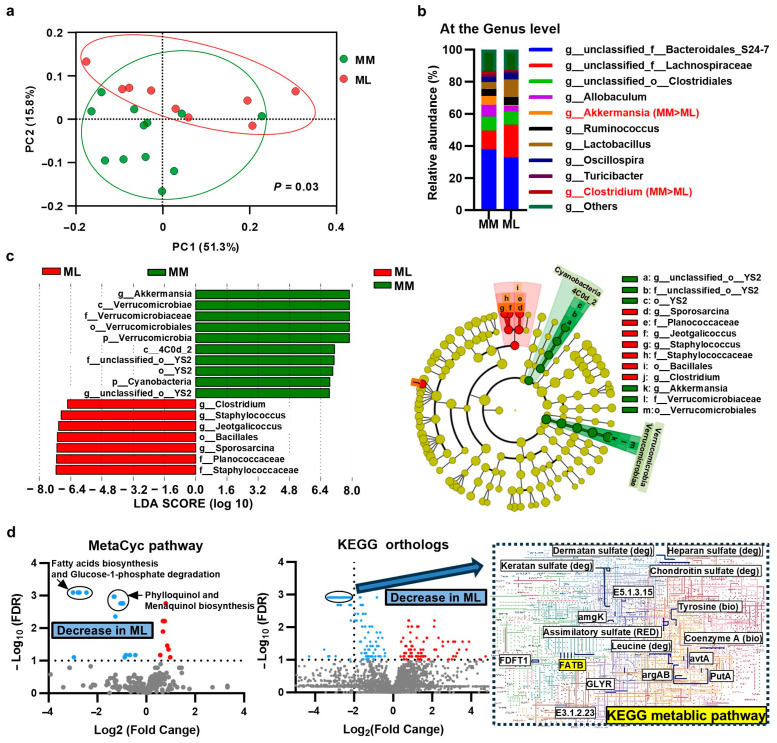
Differences in the gut microbiota (GM) composition and metabolic function between the poor muscle development (PMD) and the muscle development (MD) groups. (**a**) The weighted unique fraction (UniFrac) principal coordinate analysis (PCoA) plot of two groups. The GM showed significant differences. (**b**) The microbial taxonomic composition with the relative abundance of the two groups. The red font indicates bacterial groups that showed significant differences between the two groups. (**c**) Abundant taxa were identified using linear discriminant analysis effect size (LEfSe) analysis in each group. The abundance of beneficial *Akkermansia* bacteria was high in the MD group. (**d**) Volcano plots of differences in the GM function between the two groups based on Phylogenetic Investigation of Communities by Reconstruction of Unobserved States 2 (PICRUSt2) analysis. Fatty acid biosynthesis, glucose and glucose-1-phosphate degradation, and menaquinone biosynthesis were predicted to decrease via the MetaCyc pathway in the PMD group. Based on Kyoto Encyclopedia of Genes and Genomes (KEGG) orthologs, fatty acyl-acyl carrier protein thioesterase B (FATB), which is involved in the biosynthesis of medium-chain triglycerides, was predicted to decrease in the PMD group. The modules and kos with declined function (*q*-value < 0.001 and log2 fold change < –2.0) are indicated with blue lines on the KEGG metabolic pathway.

The microbial taxonomic composition with relative abundance at the phylum level and beta diversity is shown in Figure S2a,b. Among the GM groups, seven groups exhibited significant differences (*p =* 0.004). A heatmap of the bacterial species at the order level is shown in Figure S2c. Heatmap analysis showed homology in the GM among all groups. To assess the association between muscle mass loss and GM, the GM of the PMD and MD groups were compared. The weighted unique fraction principal coordinate analysis plot (Figure 3a) shows that the beta diversity between the two groups was significantly different (*p =* 0.003).

However, no significant differences were observed in the alpha diversity. The microbial taxonomic composition and relative abundance at the genus level are shown in Figure 3b. The relative abundance of each taxonomic group was compared at each taxonomic level between the two groups using linear discriminant analysis effect size software. Taxa with significant abundances are shown in Figure 3c. The abundance of beneficial *Akkermansia* bacteria in the *Verrucomicrobiaceae* family [22,23] and of the order *YS2* was high in the MD groups. *Sporosarcina*, *Jeotgalicoccus*, and *Staphylococcus* of Bacillales and *Clostridium* were more abundant in the PMD group than in the MD group. Furthermore, differences in GM function between the two groups were compared using PICRUSt2 analysis. Volcano plots were created to visualize the differences in important GM functions (Figure 3d). Fatty acid biosynthesis, glucose and glucose-1-phosphate degradation, and menaquinone biosynthesis were predicted to inhibit the MetaCyc pathway in the PMD group. In terms of the Kyoto Encyclopedia of Genes and Genomes (KEGG) orthologs, the fatty acyl-acyl carrier protein thioesterase B was involved in the biosynthesis of medium-chain triglycerides, and its functions were predicted to decrease in the PMD group. The modules and kos with decreased function (*q*-value < 0.001 and log2 fold change < –2.0) are shown using blue lines on the KEGG metabolic pathway (01100 M) (Figure 3d). The functional differences between the two groups are summarized in Table S1.

### 3.3. Difference in Colonic Mucosal Barrier Between PMD and MD Groups

**Figure 4:**
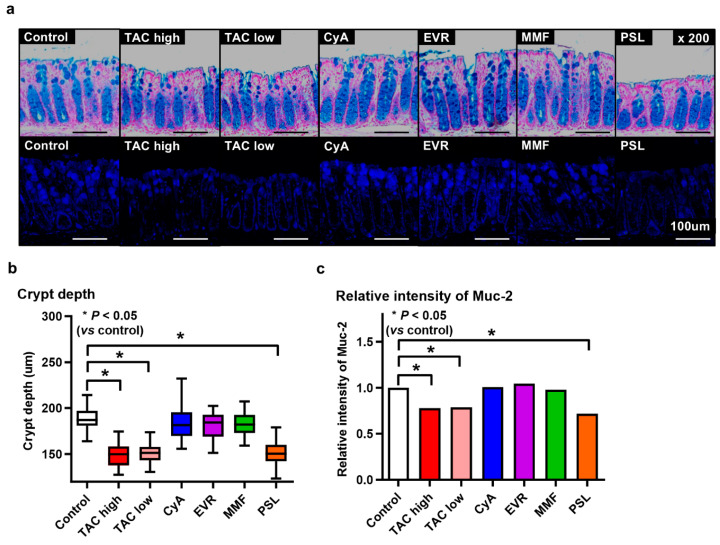
Difference in colonic mucosal barrier between poor muscle development (PMD) and muscle development (MD) groups. (**a**) Representative images of pathological specimens stained with Alcian blue and immunofluorescence of mucin-2 (MUC2). Scale bar = 100 μm. (**b**) Comparison of crypt depth in each group. (**c**) Comparison of MUC2 expression in each group.

Representative images of pathological specimens stained with Alcian blue and MUC2 immunofluorescence are shown in Figure 4a. The crypt depth (Figure 4b) and expression of MUC2 (Figure 4c) were significantly reduced in the TAC high-dose, TAC low-dose, and PSL groups (crypt depth: *p* < 0.001, *p* < 0.001, *p* < 0.001, respectively; expression of MUC2: *p =* 0.002, *p =* 0.003, *p* < 0.001, respectively), which is consistent with the groups with reduced *Akkermansia* (PMD group).

### 3.4. Patient Characteristics of KT Recipients and Donors

The demographic and clinical characteristics of the recipients and donors included in the study are summarized in Table 1. Although no significant difference was observed, the recipients tended to be younger than the donors (55 [interquartile range: 45–63] vs. 61 [interquartile range: 56–67]; *p =* 0.08) at the time of surgery. Of the 18 recipients, 13 were treated with TAC, and the remaining five received CyA. The nutritional indices (controlling nutrition status score and prognostic nutritional index) were calculated based on the lymphocyte count and serum albumin levels and were lower in the recipients (*p <* 0.001 and *p =* 0.02, respectively) than in the donors. The preoperative body composition parameters, including the psoas muscle index at the L3 level (as measured on CT images), did not differ significantly. Some preoperative body composition parameters measured using BIA differed. The total body water, protein, minerals, skeletal muscle index, and bone mineral content index were significantly higher in the recipients (*p =* 0.02, *p =* 0.04, *p =* 0.003, *p =* 0.02, *p =* 0.002, respectively) than in the donors.

### 3.5. Changes in Body Composition Parameters and Differences in GM Composition Among Preoperative and Postoperative Fecal Samples

**Figure 5:**
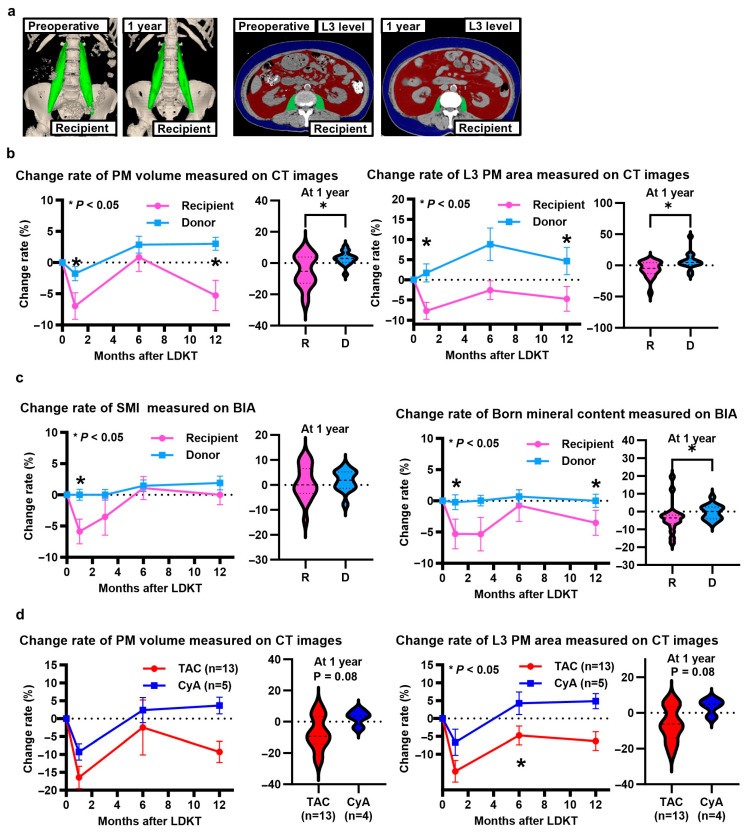
Change rate of body composition parameters measured using computed tomography (CT) images and bioelectrical impedance analysis (BIA) in recipients and donors for 1 year. (**a**) Representative CT images of recipients analyzed using SYNAPSE VINCENT. (**b**) Comparison of psoas muscle volume and psoas muscle (PM) area change rate at the L3 level between recipients and donors. (**c**) Comparison of skeletal muscle index (SMI) and bone mineral content change rate between recipients and donors based on BIA. (**d**) Comparison of psoas muscle volume and psoas muscle area change rate at the L3 level between recipients treated with tacrolimus (TAC) and cyclosporine A (CyA).

Representative CT images of the recipients, which were analyzed using SYNAPSE VINCENT to identify changes in the postoperative and preoperative body compositions between the donors and recipients, are shown in Figure 5a. The psoas muscle volume and psoas muscle area change rates at the L3 level were lower in the recipients than in the donors at 1 month and 1 year post-surgery (Figure 5b) (volume: *p <* 0.001, *p =* 0.048; area: *p =* 0.002, *p =* 0.03, respectively).

In contrast, the skeletal muscle index change rate measured using BIA did not differ significantly between the recipients and donors at 1 year (Figure 5c). Moreover, the rate of bone mineral content change measured using BIA was lower in the recipients than in the donors at 1 month and 1 year (Figure 5c).

The change rate in handgrip strength showed no difference for 1 year between the recipients and donors. A summary of the handgrip strength is listed in Table S2 (Supplementary Materials).

Furthermore, the postoperative body composition change rate (based on CT imaging) was compared between the TAC (n = 13) and CyA (n = 5) treatment groups. Although no significant difference was found, the psoas muscle volume change rate tended to be lower in the TAC group than in the CyA group at 6 months and 1 year (*p =* 0.14, *p =* 0.08, respectively) (Figure 5d). The rate of change in the psoas muscle area at the L3 level was lower in the TAC group than in the CyA group at 6 months (*p =* 0.03) and tended to be lower at 1 year (*p =* 0.08) (Figure 5d). The serum trough concentrations of calcineurin inhibitors after KT are shown in Table S3 (Supplementary Materials). The differences in the GM composition between the preoperative and postoperative fecal samples are described in the Supplementary Materials and Figure S3. Briefly, the GM of the postoperative KT group differed significantly from that of the other groups. The alpha diversity was significantly lower in the postoperative KT group than in the other two groups.

## 4. Discussion

This study revealed two important findings. First, oral TAC and PSL administration caused muscle mass loss in mice, and the GM composition in the TAC and PSL groups was significantly different from that in the other groups. Additionally, the abundance of beneficial *Akkermansia* bacteria was reduced in the PMD group, which is consistent with the histopathology results that show a decline in colon function in the PMD group. Second, muscle mass decreased 1 month following kidney transplantation, improved to the preoperative value over 6 months, and decreased again at 1 year. Additionally, the GM diversity in the postoperative fecal samples was significantly different between the recipients who were and were not administered ISs.

TAC and PSL caused muscle mass loss, and the GM composition of the TAC and PSL groups was significantly different from that of the other groups. Muscle loss is a side effect of glucocorticoids [24], and the direct catabolism of glucocorticoids occurs via the ATP– ubiquitin-dependent pathway [25]. The direct effect of calcineurin inhibitors on muscles remains controversial, although several studies have indicated that calcineurin is involved in muscle growth and wasting [26,27,28].

**Figure 6:**
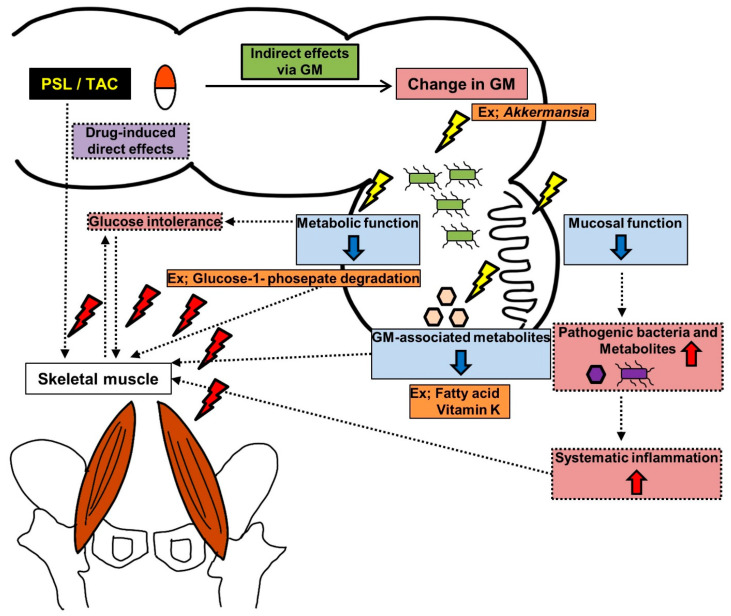
Hypothesis of mechanism underlying skeletal muscle mass loss mediated by gut microbiota (GM). The solid line indicates the mechanism suggested by this study, and the dotted lines indicate the mechanisms suggested by previous studies.

In this study, muscle atrophy was only observed in the TAC group but not in the CyA group, which cannot be solely explained by the direct effect of calcineurin inhibitors on the muscle. This suggests that the indirect effects of alteration of the GM may have influenced muscle development. GM was found to influence the muscles via the gut–muscle axis [16]. Mice devoid of a GM showed atrophy in the skeletal muscles and a decline in motor function compared with mice with a GM [29,30]. In the present study, the *Akkermansia* abundance was low in the PMD group, and colonic histopathology suggested a decline in colonic function. Additionally, PICRUSt2 analysis predicted that fatty acid biosynthesis, glucose-1-phosphate degradation, and phylloquinol and menaquinol biosynthesis would decline in the PMD group. Therefore, we hypothesized that decreased colonic mucosal function, decreased GM-associated metabolite levels, and impaired glucose tolerance due to decreased metabolic functions are factors associated with muscle mass loss (Figure 6). The mucosal barrier is an important component that maintains the intestinal barrier [31]. MUC2 is the most commonly secreted mucin by goblet cells, and the downregulated MUC2 expression observed in the PMD group suggests a reduced mucosal barrier. The importance of *Akkermansia* in intestinal mucus production and the maintenance of the intestinal barrier has been reported in mouse models of obesity, type 2 diabetes, and inflammatory bowel disease [32,33]. Fecal transplantation from young to old mice improved muscle mass, increased *Akkermansia* abundance, and enhanced mucus production [34]. Impairments in the mucosal barrier allow pathogenic bacteria and metabolites to enter other organs, leading to systemic inflammation and, ultimately, a negative impact on muscle structure and function [34]. Oral administration of *Akkermansia* improves frailty and muscle atrophy in aged mice [35]. Moreover, mucus supplementation prevents glucocorticoid-induced osteoporosis in a mice model [36]. These findings support the relationship between the mucosal barrier and muscle mass loss associated with *Akkermansia* reduction. Gut microbiota-associated metabolites play key roles in the gut–muscle axis [34]. In this study, fatty acid biosynthesis was predicted to reduce in the PMD group through the MetaCyc pathway. KEGG ortholog analysis predicted the reduction in medium-chain triglyceride biosynthesis and phylloquinol and menaquinol (also known as vitamin K) biosynthesis through the MetaCyc pathway in the PMD group. The positive effect of medium-chain triglycerides on muscle mass and function [37], the positive effects of vitamin K on muscle mass and bone [38], and the relationship between a decrease in metabolic products owing to GM alterations and muscle mass loss have been well documented. Although we did not examine bone changes in this mouse model, osteoporosis is a widely known side effect of PSL, suggesting that osteoporosis may be related to changes in GM metabolic function. Additionally, glucose intolerance due to GM alterations may be associated with muscle mass loss [39]. Reduced *Akkermansia* and vitamin K abundance is associated with glucose intolerance [38,40]. Compared with CyA, TAC is a higher risk factor for post-transplant diabetes, which is consistent with the results of this study, considering that GM alterations, glucose intolerance, and muscle atrophy are associated. However, the use of CyA in the transplantation field has decreased in recent years, and only limited reports have evaluated the effects of CyA on GM [41,42]. Therefore, the mechanism underlying the difference in the effects of TAC and CyA on muscle development is currently unknown. Probiotics and dietary modification have been highlighted as promising strategies to modulate the gut microbiota and improve metabolic outcomes in transplant recipients [41]. Although direct probiotic or oral supplementation has not been suggested for muscle loss caused by IS-induced alteration of GM changes after KT, some probiotics have been reported to improve tacrolimus-induced post-transplant adverse events through GM regulation, and it seems worth evaluating whether they contribute to improving muscle development [41,43,44]. Probiotics, oral supplementation, and medications that increase *Akkermansia* may also have a beneficial effect on the muscle after KT [35,40,45]. However, probiotics themselves have only been validated in limited clinical studies, and further investigation is needed to allow optimal IS use to improve the quality of life after KT.

In recipients, muscle mass decreased 1 month after kidney transplantation and improved to the preoperative value in the next 6 months before decreasing again at 1 year. Although preoperative frailty affects postoperative outcomes, the relationship between postoperative frailty and muscle mass remains controversial. In this study, we compared the postoperative changes in muscle mass between recipients and donors and found that muscle mass decreased in recipients despite the tendency of recipients to be younger. Previous Japanese studies have reported a consistent decrease in muscle mass [15], which is consistent with the results of this study.

Additionally, the bone mineral index followed the same trend as that of the muscle mass index. The bone mineral index was significantly low in recipients 1 year after surgery. Based on the results of the mouse experiments, we compared the recipients receiving TAC and CyA therapy. Although the difference was not statistically significant, muscle mass tended to decrease in the KT recipients undergoing TAC. The usefulness of TAC over CyA for acute rejection has been suggested [1]; hence, TAC is prescribed to many patients. However, this study suggests that CyA is a better option in selected cases to extend life expectancy, as the prevalence of older adult KT recipients increase. The GM alpha and beta diversities were significantly different in the postoperative fecal samples between the recipients who were and were not prescribed ISs (donors before and after surgery and recipients before receiving KT). Antibiotics affect GM. All postoperative recipients were prescribed a combination of sulfamethoxazole and trimethoprim to treat pneumocystis pneumonia. Therefore, the effects of sulfamethoxazole and trimethoprim on GM must be carefully considered. It is difficult to identify whether the changes in the GM after KT are due to antibiotics or the ISs. The effects of ISs on GM during antibiotic therapy should be further investigated as a potential therapeutic target.

This study has several limitations. First, the blood metabolite levels were not measured in mice or humans; therefore, the mechanisms underlying the association between GM and muscles were not elucidated. However, metabolic function in the mice was predicted using PICRUSt2 analysis. Furthermore, for direct evidence linking alterations in the GM to changes in colonic function and muscle growth, we must demonstrate that modulating the microbiota leads to corresponding changes in these physiological parameters. However, this study did not include interventions targeting the GM. Nonetheless, recent research has extensively documented the positive effects of *Akkermansia* on intestinal barrier function [46]. Consistent with these findings, our study observed changes in the colonic mucosa associated with a decrease in *Akkermansia* levels. We believe that this may be indirect evidence of linking alterations in the GM to changes in colonic function and muscle growth. Second, motor function was not evaluated in either the humans or mice. Sarcopenia is a state of muscle mass decline resulting in reduced muscle strength and physical function. Therefore, further consideration of motor function is required to evaluate sarcopenia and frailty. Finally, the sample size of the clinical study was small. Therefore, analyses of larger sample sizes are required to validate our results. Particularly, the number of human fecal samples was extremely small; hence, identifying the characteristic bacterial species of each group and performing functional analysis using PICRUSt2 was challenging. Furthermore, the number of cases available for comparison between TAC and CyA in KT recipients was low, and further prospective investigations are required.

Despite these limitations, the strength of this study lies in our examination of CyA, which has been used less frequently in recent years. We intend to conduct further studies to determine whether GM intervention improves the negative effects of ISs on muscle mass in mice. Furthermore, we hope to enroll more patients (humans) and analyze their blood metabolites.

The oral administration of TAC or PSL was associated with muscle mass loss and altered GM in mice. In a clinical study of recipients, a reduction in muscle mass was observed 1 year after kidney transplantation. Selecting appropriate calcineurin inhibitors and using probiotics, especially *Akkermansia*, might improve muscle mass loss following kidney transplantation. A better understanding of the associations among ISs, GM, and muscle mass would yield better outcomes and fewer side effects in KT recipients with frailty. Therefore, further studies are required to select and establish the optimal IS and probiotic regimen to reduce the side effects of ISs and prevent frailty.

## Figures and Tables

**Table 1:** Demographics and clinical characteristics of recipients and donors.

Variables		Recipients (n = 18)	Donors (n = 16)	*p*-Value
Preoperative demographics and clinical characteristics				
Age at surgery (years)	Median (IQR)	55 (45–63)	61 (56–67)	0.08
Sex	Male	10 (56%)	8 (50%)	0.35
	Female	8 (44%)	8 (50%)	1.00
ABO-incompatible	Yes	7 (39%)	-	
Preemptive	Yes	6 (33%)	-	
Calcineurin inhibitor	TAC	13 (72%)	-	-
	CyA	5 (28%)	-	
Antimetabolite or everolimus	MMF	18 (100%)	-	
	EVR	15 (83%)	-	-
Glucocorticoid	PSL	18 (100%)	-	
Hypertension	Yes	18 (100%)	3 (19%)	<0.001 *
Diabetes mellitus	Yes	3 (17%)	0 (0%)	0.23
Dyslipidemia	Yes	9 (50%)	2 (12%)	0.03 *
Hemoglobin (g/L)	Median (IQR)	11.3 (10.7–12.7)	13.9 (13.4–15.4)	<0.001 *
C-reactive protein (mg/dL)	Median (IQR)	0.05 (0.02–0.17)	0.03 (0.02–0.06)	0.19
Lymphocyte count	Median (IQR)	1300 (1025–1475)	1650 (1400–1900)	0.004 *
Albumin (mg/dL)	Median (IQR)	4.2 (3.8–4.6)	4.4 (4.3–4.6)	0.09
Total cholesterol	Median (IQR)	168 (154–189)	220 (199–238)	<0.001 *
CONUT score	Median (IQR)	2 (1–3)	0 (0–1)	<0.001 *
GNRI score	Median (IQR)	102 (97–106)	106 (101–110)	0.24
PNI score	Median (IQR)	48 (44–53)	52 (50–54)	0.02 *
eGFR (mL/min/1.73 m^2^)	Median (IQR)	7.3 (6.2–9.0)	85.2 (72.0–94.0)	<0.001 *
HbA1c (%)	Median (IQR)	5.7 (5.2–6.0)	5.7 (5.5–5.8)	0.83
Body weight (kg)	Median (IQR)	61.6 (54.1–72.1)	57.1 (48.7–63.7)	0.19
Height (cm)	Median (IQR)	168 (160–171)	161 (160–166)	0.17
Body mass index (kg/m^2^)	Median (IQR)	22.4 (20.0–24.8)	22.3 (19.7–23.2)	0.48
Handgrip strength (kg)	Median (IQR)	28.0 (21.3–31.4)	30.7 (24.5–34.7)	0.45
Preoperative body composition parameter measured using CT				
Psoas muscle (cm^3^)	Median (IQR)	287 (226–356)	237 (158–326)	0.22
Visceral adipose tissue (cm^3^)	Median (IQR)	1829 (1573–2896)	2533 (1597–3470)	0.57
Subcutaneous adipose tissue (cm^3^)	Median (IQR)	2629 (1985–3396)	3394 (2460–4037)	0.19
Psoas muscle index at L3 (cm^2^/m^2^)	Median (IQR)	6.2 (4.5–7.1)	4.3 (3.7–6.3)	0.15
Preoperative body composition parameter measured using BIA				
Total body water (L)	Median (IQR)	36.8 (31.0–42.3)	27.5 (26.1–35.0)	0.02 *
Protein (kg)	Median (IQR)	9.6 (8.0–11.1)	7.4 (6.9–9.5)	0.04 *
Minerals (kg)	Median (IQR)	3.5 (3.0–3.8)	2.6 (2.4–3.2)	0.003 *
Bone mineral content (kg)	Median (IQR)	2.9 (2.5–3.1)	2.2 (2.0–2.6)	0.003 *
Body fat mass (kg)	Median (IQR)	12.1 (11.2–16.0)	15.6 (12.2–17.0)	0.22
Skeletal muscle index (kg/m^2^)	Median (IQR)	7.3 (6.5–8.2)	6.0 (5.4–7.1)	0.02 *
Fat mass index (kg/m^2^)	Median (IQR)	4.6 (4.3–5.5)	5.7 (4.7–6.5)	0.11
Bone mineral content index (kg/m^2^)	Median (IQR)	1.06 (0.92–1.10)	0.87 (0.77–0.97)	0.002 *
50 kHz whole-body phase angle (°)	Median (IQR)	4.8 (3.8–5.3)	5.3 (4.4–5.9)	0.18

IQR: interquartile range, TAC: tacrolimus, CyA: cyclosporine A, MMF: mycophenolate mofetil, EVR: everolimus, PSL: prednisolone, CONUT: controlling nutritional status, GNRI: geriatric nutritional risk index, PNI: prognostic nutritional index, eGFR: estimated glomerular filtration rate, CT: computed tomography, BIA: bioelectrical impedance analysis. Categorical variables were compared using Fisher’s exact test, and continuous variables were compared using Mann–Whitney U-test. * *p*  <  0.05.

## Data Availability

The datasets generated and/or analyzed during the animal experiments are provided within the manuscript or the Supplementary Materials files. The datasets generated and/or analyzed during the current clinical study are not publicly available because of hospital policy; however, they are available from the corresponding author upon reasonable request.

## Supplementary Materials

The following supporting information can be downloaded at https://www.mdpi.com/article/10.3390/jcm14051628/s1: Supplementary information: Detailed materials and methods, including the reagents and analytical methods, part of the results of the clinical study, and supplementary figures, supplementary tables (except Table S1), references [21,47,48,49,50,51,52,53] has been cited in the Supplementary Materials file; Figure S1: Overview of data processing of 16S ribosomal RNA gene sequences; Figure S2: Differences in gut microbiota compositions owing to treatment with various immunosuppressants; Figure S3: Alterations in gut microbiota before and 1 month after surgery; Table S1: Summary of estimated functional differences between poor muscle development and muscle development groups from PICRUSt2 analysis; Table S2: Change rate of handgrip strength in recipients and donors for 1 year; Table S3: Serum trough concentrations of calcineurin inhibitors after kidney transplantation. Animal date: The datasets except gut microbiota analysis generated and/or analyzed during the animal experiments. Taxonomy: All data on the microbial taxonomic composition with relative abundance in mice. KEGG orthologs: All data for two-group comparisons (poor muscle development and muscle development groups) in the Kyoto Encyclopedia of Genes and Genomes (KEGG) orthologs generated by PICRUSt2 analysis. MetaCyc pathway: All data for two-group comparisons (poor muscle development and muscle development groups) of the MetaCyc pathway, generated by PICRUSt2 analysis.

## Abbreviations

The following abbreviations are used in this manuscript:

KT	kidney transplant
IS	immunosuppressant
GM	gut microbiota
MUC2	mucosal protein-mucin-2
ESRD	end-stage renal disease
TAC	tacrolimus
CyA	cyclosporine A
PSL	prednisolone
CT	computed tomography
IHC	immunohistochemical
BIA	bioelectrical impedance analysis
FDR	false discovery rate
PMD group	poor muscle development group
MD group	muscle development group
PICRUSt2	Phylogenetic Investigation of Communities by Reconstruction of Unobserved States 2
KEGG	Kyoto Encyclopedia of Genes and Genomes
