# Supplementary material for: Immunosuppressant-Induced Alteration of Gut Microbiota Causes Loss of Skeletal Muscle Mass: Evidence from Animal Experiments Using Mice and Observational Study on Humans
Source: J Clin Med. 2025 Feb 27;14(5):1628. doi: 10.3390/jcm14051628 (PMC11900293; doi:10.3390/jcm14051628)
Supplement: Supplementary file 1 [file jcm-14-01628-s001.zip › jcm-3430004-supplementary.pdf]

Supplementary information

Immunosuppressant-induced alteration of gut microbiota causes loss of skeletal muscle mass

Mitsuru Tomizawa[1], Shunta Hori[1], Tatsuo Yoneda[1], Fumisato Maesaka[1], Takuto Shimizu[1], Kenta Onishi[1], Yosuke Morizawa[1], Gotoh Daisuke[1], Yasushi Nakai[1], Makito Miyake[1], Kazumasa Torimoto[1], Nobumichi Tanaka[1,2], and Kiyohide Fujimoto[1*]

[1] Department of Urology, Nara Medical University, 840 Shijo-cho, Kashihara, Nara 634-8522, Japan

[2] Department of Prostate Brachytherapy, Nara Medical University, 840 Shijo-cho, Kashihara, Nara 634-8522, Japan

[*]**Corresponding author**

Kiyohide Fujimoto

Department of Urology, Nara Medical University, 840 Shijo-cho, Kashihara, Nara 634-8522, Japan

Phone: +81-744-22-3051

Fax: +81-744-22-9282

E-mail: kiyokun@naramed-u.ac.jp

Supplementary methods

Reagents

The following reagents were prepared for oral administration: tacrolimus (FK506; FUJIFILM Wako Pure Chemical Corporation, Osaka, Japan), cyclosporine A (FUJIFILM Wako Pure Chemical Corporation), everolimus (FUJIFILM Wako Pure Chemical Corporation), mycophenolate mofetil (MMF; Tokyo Chemical Industry Co., Ltd., Tokyo, Japan), and prednisolone (FUJIFILM Wako Pure Chemical Corporation). MMF was dissolved in acetone, and all other drugs were dissolved in ethanol. All solutions were diluted in a sterile saline solution, and sterile saline solution was used as the control.

Fecal sample collection, DNA extraction, and 16S ribosomal RNA (rRNA) gene sequencing

Fresh fecal samples were collected just before the procurement of samples from the mice. The fecal samples were stored on ice packs for a short time before preserving at -80 °C. DNA extraction and preparation of 16S rRNA amplicons were performed as previously described [47]. The variable V3−V4 region (341F–806R) of the 16S rRNA gene was amplified using polymerase chain reaction and subsequently subjected to high-throughput sequencing using the MiSeq paired-end sequencing system (Illumina Inc., San Diego, CA, USA). A series of DNA extraction procedures were performed by Takara Bio Co. Ltd., Shiga, Japan.

16S rRNA gene sequence processing

Data processing was performed using Quantitative Insights into Microbial Ecology 2 (QIIME2) version 2021.2 (http://qiime2.org) [48]. An overview of the data processing is provided in Fig. S1. The obtained sequences were subjected to quality filtering and denoising using the DADA2 program and QIIME2. Subsequently, the data were clustered (open reference) into operational taxonomic units (OTU) at a 99% similarity threshold using the VSEARCH program and QIIME2. Taxonomic classification was determined based on the normalized OTU table using the Naive Bayes classifier created using Greengenes reference sequences (gg_13_8, 99% clustering dataset). Barplots and heatmaps were created by calculating the relative abundances of the OTUs. To gather information on sequences related to the obtained OTU sequences, a homology search was performed using the Basic Local Alignment Search Tool included in the QIIME2 pipeline. A heatmap of the abundance ratios of hit bacterial species was created based on the homology search results. Linear discriminant analysis effect size analysis was performed using the Galaxy pipeline (http://huttenhower.sph.harvard.edu/galaxy/) to identify the characteristic taxa [49]. The OTU sequences were aligned and subjected to phylogenetic tree calculations using the QIIME pipeline. Alpha diversity (Shannon index, Chao1 index, and Faith's phylogenetic diversity)

was calculated at a sequence depth of 10,000 reads per sample with ten random iterations using QIIME2 (version 2021.2) alpha rarefaction. The weighted unique fraction (UniFrac) distances were calculated based on the OTU table and phylogenetic tree, and beta diversity was visualized based on principal coordinate analysis (PCoA) using GraphPad Prism 10.1.1 (GraphPad Software, San Diego, CA, USA). Phylogenetic Investigation of Communities by Reconstruction of Unobserved States (PICRUSt2) analysis was performed to predict the functional potential of a bacterial community based on marker gene sequencing profiles [50]. PICRUSt2 required the input sequences to be OTUs generated from closed-reference OTU-picking against a compatible version of the Greengenes database; hence, the filtered and denoised sequences were reclustered (closed reference) into OTUs. The results of the functional potential of a bacterial community from PICRUSt2 analysis are shown in MetaCyc pathways [51] and Kyoto Encyclopedia of Genes and Genomes orthologs [52].

Immunohistochemistry for mucin-2 (MUC2) and data collection

The 5-μm paraffin sections of colon tissues were incubated with MUC2 primary antibodies (rabbit; # M94132; 1:2,000; Proteintech, Rosemont, IL, USA), followed by incubation with Alexa Fluor 594 anti-rabbit immunoglobulin G secondary antibodies (cat. #711-585-152, 1:500, Thermo Fisher Scientific Inc., Waltham, MA, USA). Then, they were mounted with mounting medium containing 4',6-diamidino-2-phenylindole (Vector Laboratories, Burlingame, CA, USA). The sections were immediately examined under a fluorescence microscope (magnification: 400×; EVOS™ FL Auto Imaging System; Thermo Fisher Scientific Inc.) and photographed at the same settings. All acquired images were quantified and processed using Fiji software [53]. In each mouse, five goblet cells from well-oriented crypts were randomly selected and evaluated for expression intensity. The relative expression intensities were compared for each group. All images were converted to pseudocolor (thul) at the same settings using Fiji’s LUT function to highlight the desired features.

Body composition measurements

The unenhanced computed tomography images were analyzed using a Volume Analyzer SYNAPSE VINCENT image analysis system (Fujifilm Medical, Tokyo, Japan). Total psoas major muscle volume (cm^3), psoas major muscle area at L3 (cm^2), abdominal skeletal muscle area (cm^2), abdominal subcutaneous fat volume (cm^3), and visceral fat volume (cm^3) were obtained. Body composition was measured using multifrequency bioelectrical impedance analysis using an InBody S20® analyzer (Biospace, Tokyo, Japan), as previously described [54]. The skeletal muscle mass (kg), total body water (L), body protein mass (kg), total mineral content (kg), bone mineral content (kg), total body fat mass (kg), and 50 kHz-whole body phase angles (°) were measured automatically. The skeletal muscle mass, total body fat

mass, and bone mineral content were normalized to height (m) and defined as the skeletal muscle index, fat mass index, and bone mineral index, respectively. To evaluate changes in body composition, the preoperative change rate was calculated from the obtained data using the following formula for each patient:

(Postoperative value / Preoperative value × 100) – 100 (%).

Difference in gut microbiota (GM) composition among preoperative and postoperative fecal samples

In total, 20 fecal samples were collected from six recipients (preoperative: six samples, postoperative: four samples) and six donors (preoperative: six samples, postoperative: four samples) who had provided consent. Fecal samples were classified into four groups: before transplantation (Before Tx), after transplantation (After Tx), before donor nephrectomy (Before Nx), and after donor nephrectomy (After Nx). The beta-diversity of the GM in the four groups is shown in the weighted UniFrac PCoA plot (Fig. S3a). Among the four groups, After Tx and Before Tx (P = 0.009, q = 0.05) and After Tx and Before Nx (P = 0.018, q = 0.05) showed significant differences. The beta-diversity among fecal samples between the After Tx group exposed to ISs and other groups (No ISs: Before Tx, Before Nx, After Nx) is shown in Fig. S3b. Significant differences were observed between the groups (P = 0.001). The alpha diversity was also compared among the four groups (Fig. 3c). The Shannon and Chao1 indices were significantly lower in the After Tx than in the Before Tx, Before Nx, and After Nx groups (Shannon index: P = 0.007, q = 0.02; P = 0.03, q = 0.05; P = 0.04, q = 0.05 and Chao1 index: P = 0.007, q = 0.02; P = 0.03, q = 0.05; P = 0.04, q = 0.05, respectively). The Faith's phylogenetic diversity was lower in the After Tx group than in the Before Tx (P = 0.01, q = 0.07) group. Comparison of the alpha diversity of the fecal samples of the After Tx group exposed to ISs with that of the other groups showed that all indices were statistically lower in the After Tx group than in the other groups.

Supplementary Figures

Figure S1

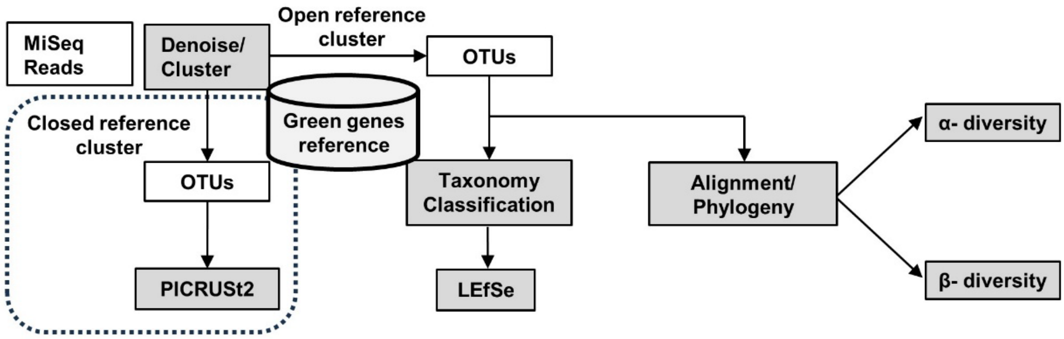

Figure S1. Overview of data processing of 16S ribosomal RNA (rRNA) gene sequences. The obtained sequences were subjected to quality filtering and denoising before clustering (open reference) into operational taxonomic units (OTUs) at a 99% similarity threshold. The taxonomy classification was determined based on the normalized OTU table according to the Naïve Bayes classifier created using Greengenes reference sequences. Barplots and heatmaps were created by calculating the relative abundance of OTUs. Linear discriminant analysis effect size (LEfSe) analysis was performed to identify the characteristic taxa. The OTU sequences were aligned and subjected to phylogenetic tree calculations in the Quantitative Insights into Microbial Ecology (QIIME) pipeline. The alpha and beta diversities were calculated based on the OTU table and the phylogenetic tree. Phylogenetic Investigation of Communities by Reconstruction of Unobserved States (PICRUSt2) requires the input sequences to be the OTUs generated from closed-reference OTU-picking against a compatible version of the Greengenes database. Hence, the filtered and denoised sequences were reclustered (closed reference) into OTUs before performing PICRUSt2 analysis.

Figure S2

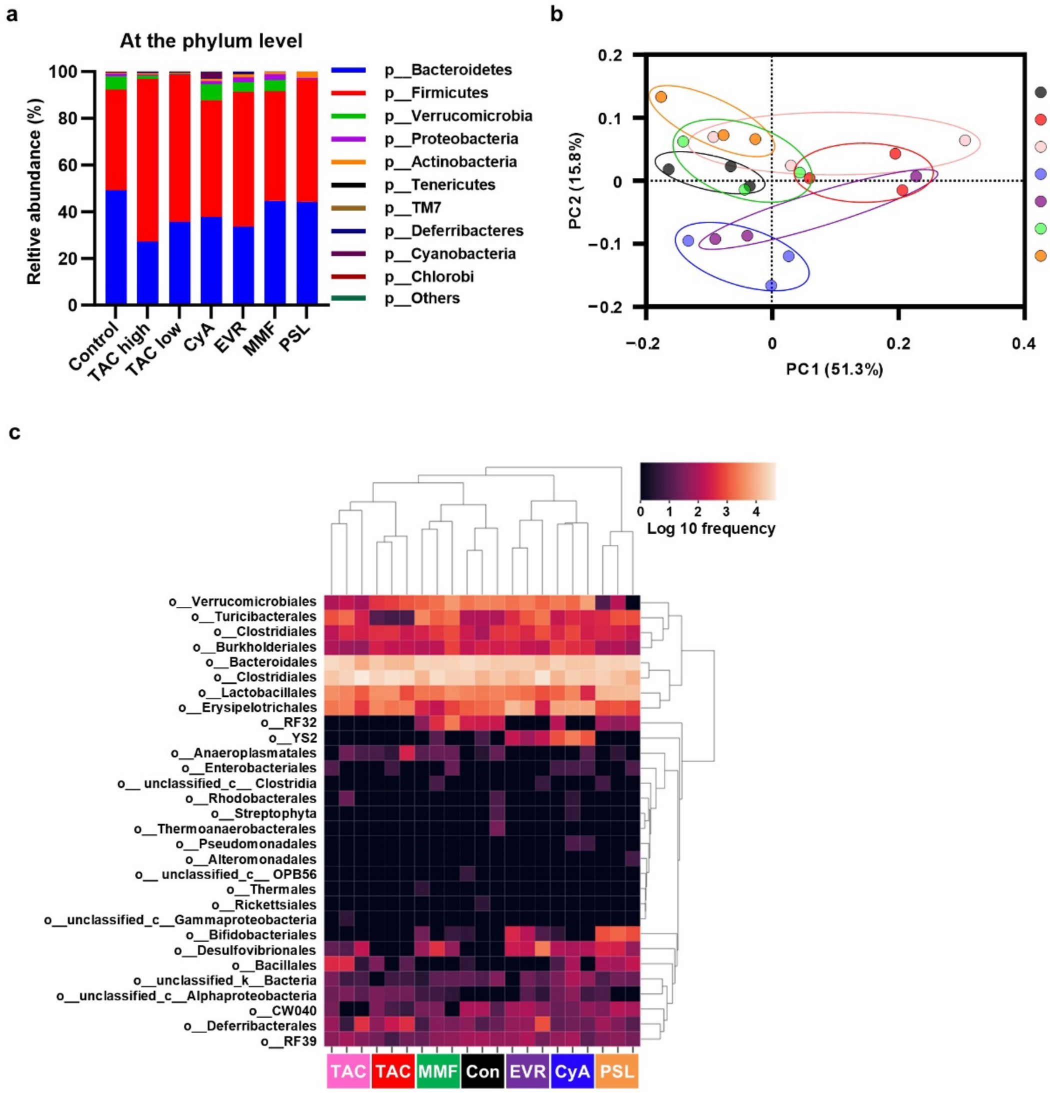

Figure S2. Difference in gut microbiota (GM) composition owing to treatment with various immunosuppressants. **(a)** Microbial taxonomic composition with the relative abundance of each group at the phylum level. **(b)** Weighted unique fraction (UniFrac) principal coordinate analysis (PCoA) plot of the GM of each group. **(c)** Heatmap of bacterial species at the order level. Heatmap analysis showed homology in the GM in each group.

Figure S3

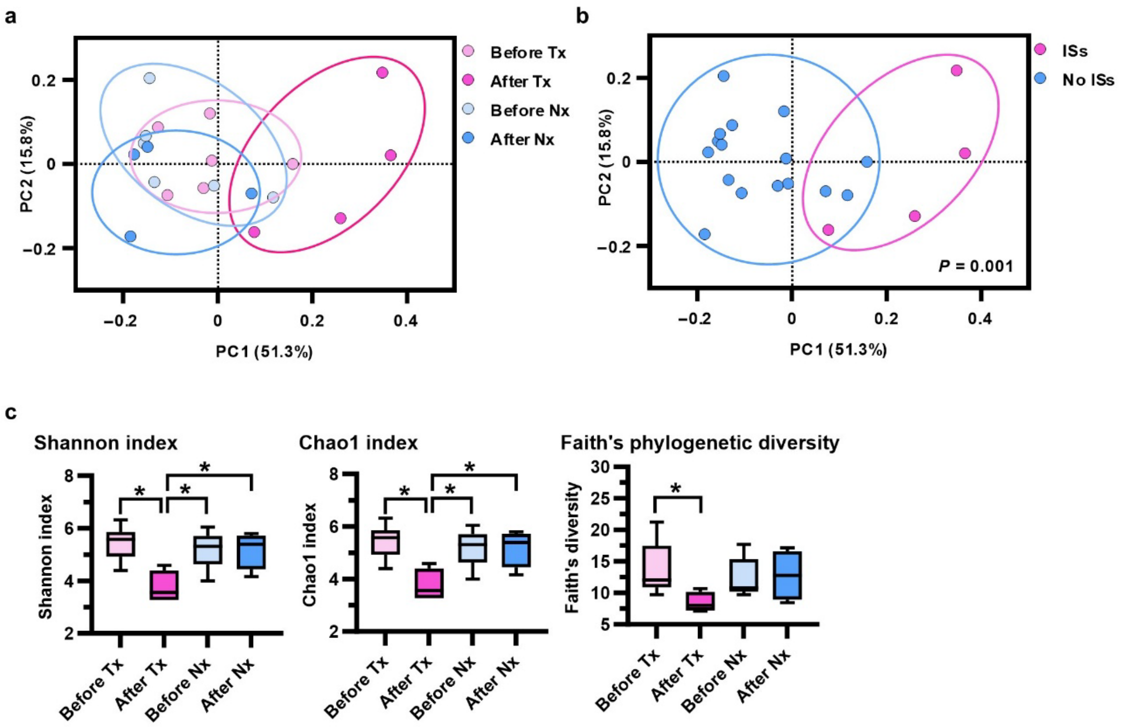

Figure S3. Alterations in gut microbiota (GM) before and 1 month after surgery. **(a)** Weighted unique fraction (UniFrac) principal coordinate analysis (PCoA) plot of four groups: before transplantation [Before Tx], after transplantation [After Tx], before donor nephrectomy [Before Nx], and after donor nephrectomy [After Nx]. **(b)** Weighted UniFrac PCoA plot comparing the fecal samples of the After Tx group exposed to immunosuppressants (IS) with that of the other groups (No ISs: Before Tx, Before Nx, and After Nx). **(c)** Comparison of the alpha diversity between the four groups.

Supplementary Tables (Table S1 is stored in a separate Excel file.)

Table S2. Change rate of handgrip strength in recipients and donors for 1 year

		Recipient (n=18)	Donor (n=16)	*P*-value
**Preoperative**				
Handgrip strength (kg)	Median (IQR)	28.0 (21.3–31.4)	30.7 (24.5–34.7)	0.45
**1 month after KT**				
Change rate (%)	Median (IQR)	-6.5 (-13.1–8.6)	-3.2 (-8.1–14.4)	0.84
**3 months after KT**				
Change rate (%)	Median (IQR)	3.5 (-7.3–7.6)	1.2 (-3.9–16.7)	0.35
**6 months after KT**				
Change rate (%)	Median (IQR)	-1.1 (-3.7–16.3)	10.1 (-1.7–15.1)	0.73
**12 months after KT**				
Change rate (%)	Median (IQR)	4.4 (-1.4–15.6)	7.6 (-1.7–17.1)	0.98

IQR = interquartile range; KT = kidney transplantation;
Continuous variables were compared using Mann-Whitney U-test.

Table S3. Serum trough concentrations of calcineurin inhibitors after kidney transplantation

		TAC (n=13)	CyA (n=5)
		**1 month after KT**	
Serum trough concentration (ng/mL)	Median (IQR)	4.9 (3.8-5.3)	180 (175-257)
Daily dosage (mg)	Median (IQR)	6 (4-8)	160 (150-300)
		**3 months after KT**	
Serum trough concentration (ng/mL)	Median (IQR)	4.6 (3.8-4.9)	129 (128-139)
Daily dosage (mg)	Median (IQR)	5 (3-7)	120 (100-300)
		**6 months after KT**	
Serum trough concentration (ng/mL)	Median (IQR)	3.7 (3.2-4.4)	108 (97-128)
Daily dosage (mg)	Median (IQR)	4 (3-7)	150 (100-250)
		**12 months after KT**	
Serum trough concentration (ng/mL)	Median (IQR)	4.8 (4.0-5.4)	111 (85-111)
Daily dosage (mg)	Median (IQR)	4 (3-7)	150 (100-200)

TAC = tacrolimus; CyA = cyclosporine A; KT = kidney transplantation; IQR = interquartile range

References

21. Miyake, M.; Morizawa, Y.; Hori, S.; Marugami, N.; Iida, K.; Ohnishi, K.; Gotoh, D.; Tatsumi, Y.; Nakai, Y.; Inoue, T.; et al. Integrative assessment of pretreatment inflammation-, nutrition-, and muscle-based prognostic markers in patients with muscle-invasive bladder cancer undergoing radical cystectomy. *Oncology* **2017**, *93*, 259–269. DOI:10.1159/000477405.

47. Matsuki, T.; Watanabe, K.; Fujimoto, J.; Takada, T.; Tanaka, R. Use of 16S rRNA gene-targeted group-specific primers for real-time PCR analysis of predominant bacteria in human feces. *Appl. Environ. Microbiol.* **2004**, *70*, 7220–7228. DOI:10.1128/AEM.70.12.7220-7228.2004.

48. Bolyen, E.; Rideout, J.R.; Dillon, M.R.; Bokulich, N.A.; Abnet, C.C.; Al-Ghalith, G.A.; Alexander, H.; Alm, E.J.; Arumugam, M.; Asnicar, F.; et al. Reproducible, interactive, scalable and extensible microbiome data science using QIIME 2. *Nat. Biotechnol.* **2019**, *37*, 852–857. DOI:10.1038/s41587-019-0209-9.

49. Segata, N.; Izard, J.; Waldron, L.; Gevers, D.; Miropolsky, L.; Garrett, W.S.; Huttenhower, C. Metagenomic biomarker discovery and explanation. *Genome Biol.* **2011**, *12*, R60. DOI:10.1186/gb-2011-12-6-r60.

50. Douglas, G.M.; Maffei, V.J.; Zaneveld, J.R.; Yurgel, S.N.; Brown, J.R.; Taylor, C.M.; Huttenhower, C.; Langille, M.G.I. PICRUSt2 for prediction of metagenome functions. *Nat. Biotechnol.* **2020**, *38*, 685–688. DOI:10.1038/s41587-020-0548-6.

51. Caspi, R.; Billington, R.; Fulcher, C.A.; Keseler, I.M.; Kothari, A.; Krummenacker, M.; Latendresse, M.; Midford, P.E.; Ong, Q.; Ong, W.K.; et al. The MetaCyc database of metabolic pathways and enzymes. *Nucleic Acids Res.* **2018**, *46*, D633–D639. DOI:10.1093/nar/gkx935.

52. Kanehisa, M.; Goto, S. KEGG: Kyoto encyclopedia of genes and genomes. *Nucleic Acids Res.* **2000**, *28*, 27–30. DOI:10.1093/nar/28.1.27.

53. Schindelin, J.; Arganda-Carreras, I.; Frise, E.; Kaynig, V.; Longair, M.; Pietzsch, T.; Preibisch, S.; Rueden, C.; Saalfeld, S.; Schmid, B.; et al. Fiji: an open-source platform for biological-image analysis. *Nat. Methods.* **2012**, *9*, 676–682. DOI:10.1038/nmeth.2019.
